# Clinical Relevance of Anti-C3 and Anti-C4 Autoantibodies in Lupus Nephritis

**DOI:** 10.1016/j.ekir.2024.01.052

**Published:** 2024-02-02

**Authors:** Vasil Vasilev, Mikel Rezola Artero, Marijana Petkova, Galya Mihaylova, Marie-Agnes Dragon-Durey, Maria Radanova, Lubka T. Roumenina

**Affiliations:** 1 Clinic of Nephrology, University Hospital - “Tzaritza Yoanna – ISUL”, Medical University of Sofia, Sofia, Bulgaria; 2 Centre de Recherche des Cordeliers, INSERM, Sorbonne Université, Université de Paris, Team Inflammation, complement and cancer, Paris, France; 3 Department of Bacteriology and Immunology, Haartman Institute, and Translational Immunology Research Program, University of Helsinki, Helsinki, Finland; 4 Department of Biochemistry, Molecular Medicine and Nutrigenomics, Medical University of Varna, Varna, Bulgaria; 5 Laboratoire d'Immunologie, Hôpital Européen Georges Pompidou, APHP, Paris, France; Université de Paris, Paris, France

**Keywords:** anti-C3 autoantibodies, anti-C3b autoantibodies, anti-C4 autoantibodies, biomarkers, complement, lupus nephritis

## Abstract

**Introduction:**

Complement system overactivation is pivotal in lupus nephritis (LN) pathophysiology. Considering that anti-C3 autoantibodies play a significant role in LN pathophysiology, we explored them as disease activity biomarkers and compared them to the ones against the homologous protein, C4.

**Methods:**

We investigated the presence of anti-C3 and anti-C4 IgG autoantibodies in a LN cohort (*N* = 85 patients) and monitored their changes over time. We correlated autoantibody presence with clinical parameters. We conducted cross-sectional and longitudinal analyses (*N* = 295 samples, 8 years follow-up) to explore associations between autoantibodies and disease progression. Antigen-specific anti-C3 or anti-C4 IgG were purified from plasma by affinity chromatography and their reactivity was tested for cross-reactivity against purified C3 or C4 by enzyme-linked immunosorbent assay (ELISA).

**Results:**

The reactivity against C3 was independent of C4. Our study revealed distinct roles for anti-C3 and anti-C4 in LN. Anti-C3 IgG exhibited stronger clinical correlations than anti-C4, showing associations with hypocomplementemia, anti-dsDNA, class IV LN, and active disease according to British Isles Lupus Assessment Group (BILAG) renal score. In a longitudinal analysis, anti-C3 positivity at initial sampling predicted present and future disease exacerbation alone and even better when combined with anti-dsDNA, as indicated by a transition to BILAG category A.

**Conclusion:**

Our research provides insights into anti-C3/C3b and anti-C4 autoantibodies in LN, revealing that they are often not cross-reactive. Anti-C3 utility as disease activity biomarkers is underscored by its stronger clinical associations and predictive value for future flares. Combining anti-C3 and anti-dsDNA out-performs the 2 factors alone, suggesting that the incorporation of anti-C3/C3b quantification into routine clinical practice could improve LN management.

## Graphical abstract

**Figure:**
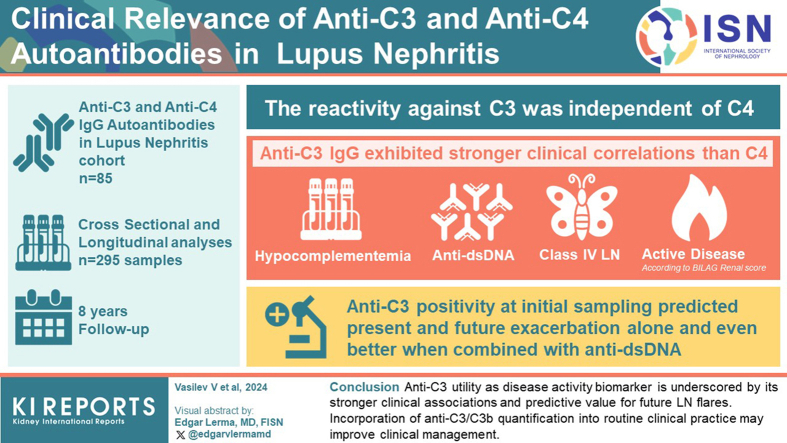

The management of a patient with LN relays in the accurate distinction between active nephritis and chronic condition.^1^ Disease activity biomarkers, focused on the innate immune complement system, hold promise in this respect; nevertheless, reliable biomarkers predicting flares are still lacking.

Complement deposition on abundant immune complexes found in the kidney contributes to tissue damage^2^; however, the mechanisms behind the enhancement of the complement activation in LN are not fully understood. IgG autoantibodies, targeting complement C1q and C3 and perturbing their functions are described in LN.^3^ Anti-C3 (or anti-C3b) autoantibodies inhibit C3b interaction with its major regulatory proteins, FH and CR1, thus leading to complement overactivation.^4^^,^^5^ These antibodies occur in over 30% of patients with LN, have higher frequency in active disease, are absent in the normal population, and are very rare in patients with systemic lupus erythematosus without LN, in C3 glomerulopathies (C3G) or other renal diseases.^1^^,^^3^, ^4^, ^5^, ^6^, ^7^, ^8^, ^9^, ^10^, ^11^, ^12^, ^13^, ^14^, ^15^ In contrast to anti-C3/C3b, anti-C4 autoantibodies have not been systematically explored and their clinical relevance is not sufficiently explained. C3 and C4 share high structural and sequence homology; however, it is still unclear whether the anti-C3 cross-react on common epitope with C4 or come from independent B cell clones. Therefore, we aimed to explore the clinical relevance of anti-C3 and anti-C4 autoantibodies in LN.

Here, we report elevated anti-C3 and anti-C4 IgG in patients with LN. Even though they may cooccur in the same patient, anti-C3 often do not cross-react with C4 and vice versa, suggesting that they originate from independent B cell clones. Anti-C3 demonstrated superior clinical relevance over anti-C4. Anti-C3 combined with anti-dsDNA is a better biomarker than each factor alone for disease activity and predictors of LN flares.

## Methods

### LN Patient Cohort

This single-center study involved 85 adult patients with systemic lupus erythematosus, clinically diagnosed following the criteria of the American College of Rheumatology. EDTA-plasma for autoantibody exploration was collected at every visit of the enrolled patients and from 28 healthy donors.

The renal score of the BILAG was used to determine the activity of LN.^16^ This scoring system is used to assess clinical disease activity and categorizes patients into distinct groups according to the intended treatment approach by physicians. The patients were grouped into 4 BILAG categories as follows: (i) category A LN represented active disease requiring disease-modifying treatment (*n* = 26, 30.6%), (ii) category B LN indicated less active disease than Category A, requiring symptomatic treatment (*n* = 26, 30.6%), (iii) category C LN indicated stable mild disease (*n* = 5, 5.9%), and (iv) category D LN indicated inactive disease (*n* = 28, 32.9%).

The study spanned 8 years, and 85 patients were followed-up with a total of 295 plasma samples. At enrollment, the cohort had a median age of 43.8 (interquartile range [IQR]: 34.5–57.0) years (from 19 to 87) and a median LN duration of 9.0 (IQR: 1.6–16.5) years (from 0.02 to 41), and 68 patients were female (80%) (Table 1, Supplementary Table S1).

**Table 1:** Demographic, clinical, immunological, and histological characteristics of patients with lupus nephropathy according to anti-C3 and anti-C4 status at first sampling

Characteristics	All patients (*N* = 85)	Only anti-C3 positive (*n* = 12)	Only anti-C4 positive (*n* = 13)	Anti-C3 and Anti-C4	
				Positive (*n* = 11)	Negative (*n* = 49)
Age^a^, median yr. (IQR)	43.0 (35.0–57.0)	34.5 (29.3–43.5)	52.0 (38.0–58.0)	32.0 (27.0–39.0)	45.0 (37.0–59.0)
Female sex, no. (%)	68 (80.0)	9 (75.0)	11 (84.6)	10 (90.9)	38 (77.6)
Duration of LN, median yr. (IQR)	9.0 (1.6–16.5)	8.5 (3.4–11.8)	13.0 (9.0–22.5)	4.0 (0.5–11.0)	6.0 (1.1–17.0)
Proteinuria, Median g/24h (IQR)	0.45 (0.10–1.60)	1.26 (0.27–2.09)	0.08 (0.05–2.60)	1.11 (0.10–5.73)	0.37 (0.10–1.01)
Serum Creatinine, median μmol/l (IQR)	68 (58–91)	72 (60–137)	71 (59–90)	64 (55–74)	69 (58–93)
CKD stage according to eGFR^b^ no. (%)					
G1	44 (51.8)	7 (58.4)	6 (46.2)	8 (72.7)	23 (46.9)
G2	25 (29.4)	1 (8.3)	5 (38.4)	2 (18.2)	17 (34.7)
G3a	5 (5.9)	1 (8.3)	1 (7.7)	1 (9.1)	2 (4.1)
G3b	4 (4.7)	2 (16.7)	0 (0.0)	0 (0.0)	2 (4.1)
G4	5 (5.9)	1 (8.3)	1 (7.7)	0 (0.0)	3 (6.1)
G5	2 (2.3)	0 (0.0)	0 (0.0)	0 (0.0)	2 (4.1)
Pathological urinary sediment^c^ (no. (%))	42 (48.3)	6 (50.0)	6 (46.2)	8 (72.7)	21 (42.9)
C4 complement^d^, median g/l (IQR)	0.22 (0.12–0.30)	0.14 (0.07–0.21)	0.22 (0.14–0.27)	0.09 (0.07–0.18)	0.27 (0.15–0.34)
C3 complement^d^^,^ median g/l (IQR)	1.14 (0.93–1.45)	1.00 (0.45–1.22)	1.12 (0.93–1.41)	0.79 (0.59–1.11)	1.24 (1.01–1.52)
Anti-dsDNA, median U/ml (IQR)	14.8 (7.7–42.5)	40.1 (16.7–79.6)	17.4 (4.5–42.2)	28.0 (15.8–195.8)	9.9 (6.6–16.1)
Anti-C1q^e^, median NU (IQR)	0.35 (0.13–0.88)	0.67 (0.25–1.94)	0.15 (0.08–0.51)	0.49 (0.20–1.46)	0.37 (0.14–0.65)
Activity of LN according to BILAG renal score^f^, no. (%)					
A	26 (30.6)	6 (50.0)	4 (30.8)	6 (54.5)	10 (20.4)
B	26 (30.6)	1 (8.3)	4 (30.8)	2 (18.2)	20 (40.8)
C	5 (10.6)	2 (16.7)	0 (0.0)	0 (0.0)	7 (14.3)
D	28 (27.0)	3 (25.0)	5 (38.4)	3 (27.3)	12 (24.5)
E	0 (0.0)	0 (0.0)	0 (0.0)	0 (0.0)	0 (0.0)
Biopsy proven histology of LN, no. (%)	80 (94.1)	11 (91.7)	12 (92.4)	10 (90.9)	47 (95.9)
Class I	4 (4.7)	0 (0.0)	1 (7.7)	0 (0.0)	3 (6.1)
Class II	25 (29.4)	3 (25.0)	3 (23.1)	2 (18.2)	17 (34.7)
Class III	8 (9.4)	0 (0.0)	2 (15.4)	1 (9.1)	5 (10.2)
Class IV	29 (34.1)	6 (50.0)	4 (30.8)	6 (54.5)	13 (26.5)
Class V	13 (15.3)	2 (16.7)	1 (7.7)	1 (9.1)	9 (18.4)
Class VI	1 (1.2)	0 (0.0)	1 (7.7)	0 (0.0)	0 (0.0)
Histology activity index^g^, median (IQR)	3.0 (1.0–5.0)	3.0 (2.5–9.8)	2.0 (0.8–3.5)	5.0 (1.5–5.8)	3.0 (1.0–5.0)
Histology chronicity index^g^, median (IQR)	1.0 (0.0–3.0)	2.5 (0.8–5.3)	2.0 (0.8–4.8)	1.0 (0.0–2.0)	1.0 (0.0–3.0)
Current immunosuppressive therapy, no. (%)	56 (65.9)	10 (83.3)	6 (46.2)	10 (90.9)	30 (61.2)
Previous Therapy with^h^: no./median months (infusions, sessions) (IQR)					
CS	81/28.0 (13.0–78.5)	12/38.0 (18.0–106.0)	13/36.0 (24.0–102.0)	9/42.0 (15.0–90.0)	47/24.0 (11.0–60.0)
CYC	72/9.5 (4.3–12.0)	8/8.0 (6.3–10.0)	12/10.0 (8.5–10.0)	8/8.5 (2.3–15.0)	44/8.0 (3.0–12.0)
AZA/MMF	41/10.0 (3.5–14.0)	8/12.0 (8.8–41.5)	5/10.0 (4.0–23.0)	5/8.0 (6.0–16.0)	23/8.0 (2.0–12.0)
CyA	17/6.0 (4.0–8.0)	3/6.0 (6.0–12.0)	3/6.0 (3.0–10.0)	1/6.0	10/6.0 (3.0–9.0)
IVIG	9/3.0 (1.0–4.5)	4/4.5 (1.5–6.8)	1/3.0	0/0.0	4/1.5 (1.0–2.8)
Plasmapheresis	23/5.0 (3.0–6.0)	3/6.0 (6.0–10.0)	9/5.0 (4.0–8.0)	4/2.5 (2.0–4.5)	7/4.0 (2.0–5.0)
History of remission in the past no. (%)	45 (52.9)	5 (41.7)	9 (69.2)	4 (36.4)	27 (55.1)
Median duration of current remission, median months (IQR)	13.0 (6.0–48.0)	6.0 (5.0–12.0)	72.0 (11.5–216.0)	36.0 (10.0–48.0)	18.0 (3.3–34.5)

AZA, azathioprine; BILAG, British Isles Lupus Assessment Group; CKD, chronic kidney disease; CS, corticosteroids; CyA, cyclosporine A; CYC, cyclophosphamide; eGFR, estimated glomerular filtration rate; IQR, interquartile range; IVIG, intravenous immunoglobulin G; LN, lupus nephritis; MMF, mycophenolate mofetil.

a The age of patients is presented as median years (yr.) and interquartile range (IQR).

b CKD stages according to eGFR are defined as follows: G1: eGFR ≥90 ml/min per 1.73 m^2^; G2: eGFR from 89 to 60 ml/min per 1.73 m^2^; G3a: from 59 to 45 ml/min per 1.73 m^2^; G3b: from 44 to 30 ml/min per 1.73 m^2^; G4: from 29 to 15 ml/min per 1.73 m^2^; and G5: <15 ml/min per 1.73 m^2^. eGFR calculated using the CKD-Epidemiology Collaboration Creatinine 2009 formula. The quantitative data are presented as absolute number of patients and percentage of total number of patients (no. [%]).

c Pathological urinary sediment is defined as the presence of more than 5 erythrocytes and more than 5 leukocytes per microliter of uncentrifuged urine.

d Normal values for C3 ranged between 0.75 and 1.65 g/l, whereas those for C4 ranged between 0.20 and 0.65 g/l.

e The Anti-C1q levels are presented as median value in normalized units (NU) and IQR. NU is defined as the ratio between the patient’s specific value of optical density at the wavelength of the optical analyzer 450 ηm and the determined cut-off for the autoantibody.

f Patients are categorized according to BILAG renal score at the time of plasma sampling. BILAG renal score assesses the activity of lupus nephropathy according to the presence of clinical, laboratory, and histology features. BILAG renal score category A means that lupus nephropathy is active and requires steroid and/or immunosuppressive treatment; category B means that the activity of lupus nephropathy requires only symptomatic treatment; category C means that LN is mild and stable condition; category D means previous renal disease without activity; and category E indicates no previous renal disease.

g The histological activity index and histological chronicity index in patients are determined according to The National Institute of Health and are presented as median value and IQR.

h The data are presented as number of patients treated and median duration (for CS, CYC, AZA/MMF) in months and median courses (for IVIG and plasmapheresis) with IQR.

Patients with LN whose diagnosis was confirmed through renal biopsy were categorized based on the LN classification of the International Society of Nephrology and the Renal Pathology Society^17^ (Table 1). The histological indices of activity and chronicity according to The National Institute of Health were determined (Table 1). Only biopsies performed less than 12 months before or after the time of autoantibody sampling were included in the correlation analysis with histological findings.

Positivity for antinuclear antibody was determined using indirect immunofluorescence on Hep-2 cells, whereas levels of anti-dsDNA were measured via ELISA (expressed in U/ml). The plasma concentration of complement components C4 and C3 was assessed using radial immunodiffusion.

The study was approved by the Ethics Review Boards of Medical University of Varna (protocol No.62/04 May 2017). Each participant signed an informed consent for the enrolment and the study was conducted following the guidelines of the Declaration of Helsinki.

### ELISA for Detecting Anti-C3 and Anti-C4 Autoantibodies

The levels of anti-C3 and anti-C4 IgG were compared in healthy donors versus patients with LN by ELISA, using established protocols.^5^^,^^15^ ELISA plates were coated with 20 μg/ml purified C3 or C4 (Complement Technology, Tyler, TX) in PBS for 1 hour. Blocking was done with PBS-0.25% Tween20. The EDTA plasma samples were then diluted 1:100 in PBS-0.05% Tween20 and were added to the plates for 1 hour. The IgG that bound to the plates was detected by anti-human IgG-HRP (Southern Biotech, 1:1000 dilution in PBS-0.05% Tween20). Reaction was revealed by 3,3′,5,5′-tetramethylbenzidine substrate and stopped with 1M H_2_SO_4_. The normal range (the cut-off of positivity) was determined as mean+2SD of 80 healthy donors. Healthy donors were tested in each run and the titers were determined as fold change compared to the mean of the healthy donors, which was set as 1.

### Cross-Reactivity Assessment

IgG was purified from plasma of patients with LN, positive for both anti-C3 and anti-C4 reactivity (*n* = 5) or from healthy donors (*n* = 2) by Protein G beads (GE Healthcare, Vélizy, France). The concentration of IgG protein was quantified using Nanodrop. The purity of the obtained IgG samples was assessed through SDS-PAGE electrophoresis on 10% v/v precast polyacrylamide gels (Thermo Fisher Scientific, Illkirch-Graffenstaden, France).

For the immunodepletion of the purified IgG samples from reactivity against C3 or C4, CNBr-activated sepharose 4B (Cytiva, France) beads were coupled with purified C3 or C4 (Complement Technology, Tylor, TX) at a concentration of 5 mg/ml and prepared for affinity chromatography. Purified IgG from patients positive for both anti-C3 and anti-C4 and from healthy volunteers (1 mg/ml) were passed through the columns to deplete IgG with reactivity to either C3 or C4. The eluate from each column was collected and analyzed for reactivity to immobilized C3 or C4 by ELISA.

### *In Silico* Prediction of the Potential B Cell Epitopes, Common Between C3 and C4

Sequence alignment of C3 and C4 was performed by Uniprot ClustalO (https://www.expasy.org/resources/uniprot-clustalo). The B cell epitopes were predicted using the IEBD server (https://www.iedb.org/). Structure superposition of C3b and C4b was done by the United States Geological Survey Chimera software (https://www.cgl.ucsf.edu/chimera/download.html). The position of the predicted common epitopes on C3b and on C4b structures was visualization done by PyMol (https://pymol.org/2/).

### Statistical Analyses

Software R studio (R Foundation for Statistical Computing, Vienna, Austria) and GraphPad Prism (La Jolla, CA) were used to generate statistical analyses. Association between 2 categorical variables was evaluated by a 2-sided Fisher exact test and association between a categorical variables and continuous variables by Wilcoxon rank sum test. The R software version 4.2.2 was used to generate the tables, Kaplan-Meier curves, receiver operating characteristic curves, Cox regression and Logistic regression analyses with the “finalfit” and “survival” packages. A log-rank test was applied to examine the survival difference between groups stratified according to antibody positivity calculated as mean ± 2SD of the normal range.

## Results

### Anti-C3 and Anti-C4 Autoantibodies Frequency in LN Cohort

**Figure 1:**
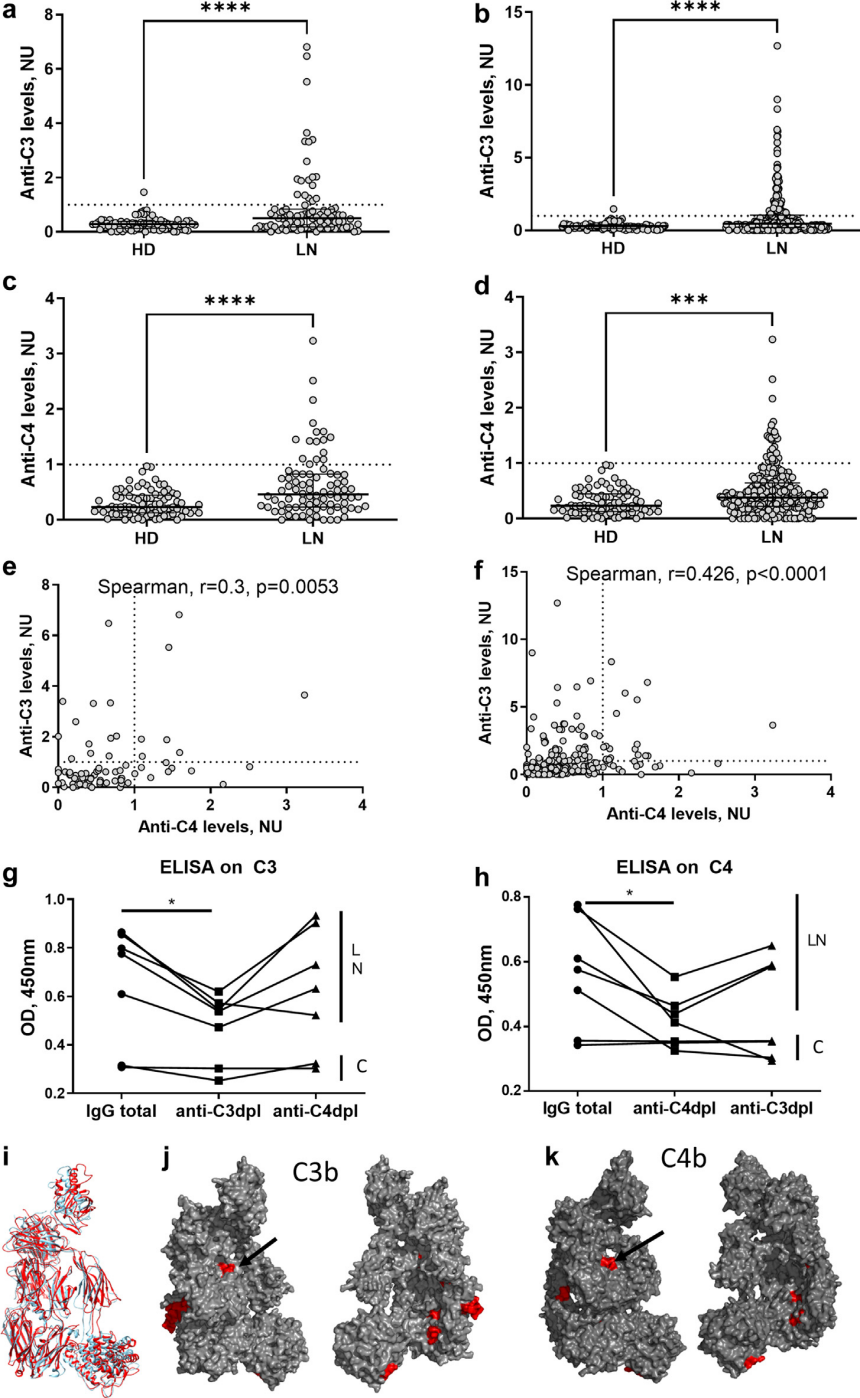
Autoantibodies against C3 and against C4 in patients with LN. Titers of anti-C3 autoantibodies in patients with LN compared to healthy donors (HD) (a) at cross-section, at first available sampling and (b) in all available follow-up samples. Titers of anti-C4 autoantibodies (c) at cross-section and (d) in all available follow-up samples. The comparison was done using Mann-Whitney test. Correlation between the titers of anti-C3 and anti-C4 (e) at cross-section and (f) in dynamics in all available samples. Spearman correlation test. (g and h) Evaluation of the cross-reactivity of anti-C3 and anti-C4 autoantibodies. IgG, purified from double-positive LN patients’ plasma or from healthy donors by protein G, was depleted from reactivity against C3 or C4 by affinity chromatography on sorbent-immobilized C3 or C4 respectively. The total IgG, as well as anti-C3 and anti-C4 depleted IgG fractions were probed for reactivity against immobilized (g) C3 or (h) C4 using enzyme-linked immunosorbent assay. The black lines link the values obtained for the IgG preparations derived from the same donor. Wilcoxon matched-pairs signed rank test. LN – patients, C – healthy controls. (i–k) *In silico* evaluation of the B cell epitopes of C3b and C4b. (i) Strong structural homology between C3b (red) and C4b (blue) Structure superposition done by United States Geological Survey Chimera software. Position of the predicted common epitopes (red) on (j) C3b and on (k) C4b. Visualization done by PyMol. HD, healthy donors; LN, lupus nephritis.

We detected anti-C3 and anti-C4 autoantibodies in 23 (27.1%) and 24 (28.2%), respectively, of the patients with LN in a cross-sectional analysis (of which both anti-C3 and anti-C4 was elevated in 11 [12.9%] of the patients) (Table 1, Figure 1a and c). Longitudinal study showed elevated anti-C3 and anti-C4 IgG in 89 (30.2%) and 53 (18.0%) patients, respectively, of the samples (with both anti-C3 and anti-C4 elevated in 28 [9.5%] of the samples) (Figure 1b and d). There was a weak but significant correlation between the anti-C3 and anti-C4 titers at cross-section (Spearman, r = 0.3; *P* < 0.0053) and in dynamics (Spearman, r = 0.426; *P* < 0.0001) (Figure 1e and f). Anti-C3 were found in younger patients, whereas anti-C4 did not correlate with age (Supplementary Table S1).

### Anti-C3 and Anti-C4 in LN Most Often are Not Cross-Reactive

The discovery of double positive anti-C3 and anti-C4 patients raised the question whether this was due to cross-reactivity of the same antibodies with these proteins, or due to concomitant existence of IgGs derived from different B cell clones. We probed the reactivity against immobilized C3 or C4 of total IgG, as well as anti-C3 and anti-C4 depleted IgG fractions from the same patients using ELISA. The reactivity against both C3 and C4 was high in the total IgG. The signal decreased when the IgGs were passed over C3-functionalized or C4-functionalized sorbent and probed against the same protein, validating the partial immunodepletion. When anti-C4-depleted IgG were probed against immobilized C3, 4 of 5 tested samples retained their original reactivity and in only 1 case, the reactivity was lost, supporting a cross-reactivity phenomenon (Figure 1g). When the anti-C3-depleted IgG were reacted with immobilized C4, 3 of 5 samples retained their original reactivity, and for 2, the signal decreased, including the one derived from the same patient, which lost reactivity in the C3 test (Figure 1h). These results suggest that in the majority of cases, the autoantibodies epitopes are independent, even though at least 1 case presented clear cross-reactivity.

Immobilized C3 and C4 behave like C3b and C4b in their binding properties. Despite the strong structural homology between C3/C3b and C4/C4b (Figure 1i), which would suggest existence of common epitopes, the sequence identity between C3 and C4 is only 25% with 466 identical positions and 541 similar positions. Although some of the predicted B cell epitopes overlapped on the structure of C3b and C4b, only 4 predicted epitopes shared more than 50% identical sequence (Figure 1j and k).

### Anti-C3 but Not Anti-C4 Autoantibodies Associate With Hypocomplementemia

**Figure 2:**
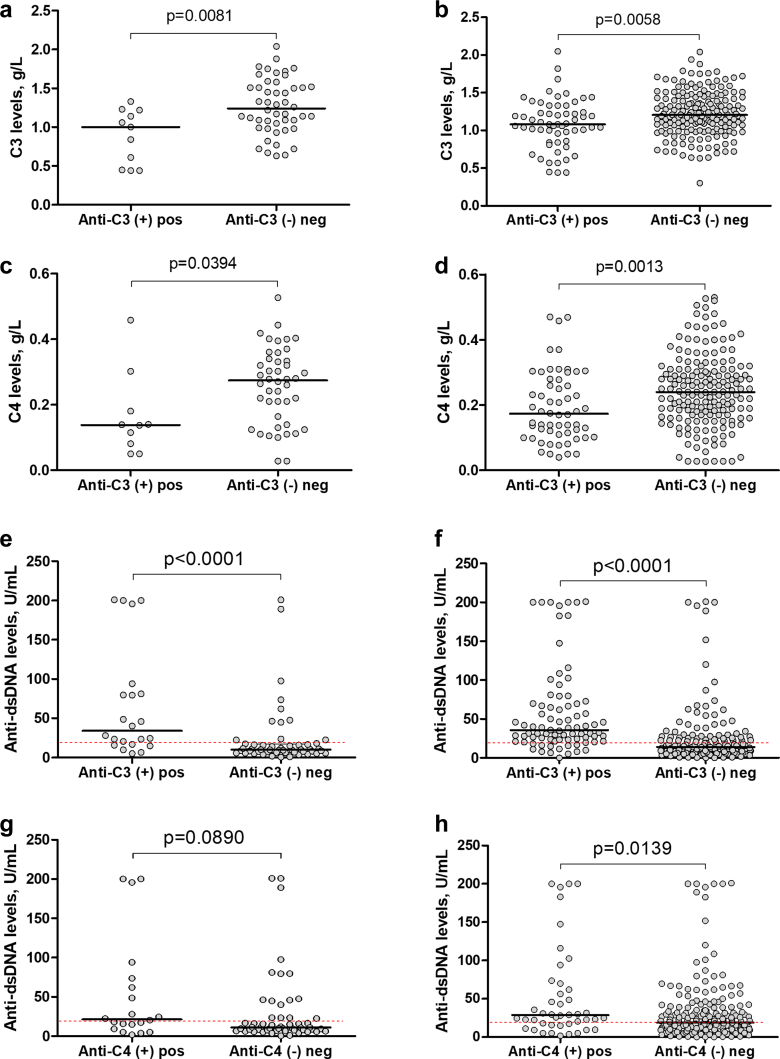
Association of anti-C3 and anti-C4 autoantibodies with immunological markers of LN activity. Association of anti-C3 with: (a) C3 in cross-section, (b) C3 in dynamic, (c) C4 in cross-section, and (d) C4 in dynamic. Association of anti-dsDNA with: anti-C3 (e) in cross-section, (f) in dynamic, or anti-C4 (g) in cross-section, (h) in dynamic (Mann-Whitney test). The red dashed lines indicate the lower reference limits for C3 (0.75 g/l) and C4 (0.20 g/l), respectively; and the upper reference limit for anti-dsDNA levels (20 U/ml). neg, negative; pos, positive.

One *in vivo* argument for potential functional consequences of the anti-C3 or anti-C4 could be their correlation with C3 and/or C4 consumption. C3 levels in patients positive for anti-C3 (median: 1.000 g/l, IQR: 0.450–1.220) at first sample were lower than C3 levels in negative ones (median: 1.240 g/l, IQR: 1.005–1.518) (Mann-Whitney, *P* = 0.0081) (Figure 2a). The same difference was present in the C3 levels found among all tested samples in patients monitored in dynamics: C3 levels in samples positive for anti-C3 (median: 1.080 g/l, IQR: 0.860–1.330) were lower than C3 levels in negative ones (median: 1.205 g/l, IQR: 1.043–1.433) (*P* = 0.0058) (Figure 2b). For 7 out of 12 patients positive for anti-C3 at baseline, more than 1 plasma sample was available over time. In 5 of these 7 anti-C3 positive and followed-up patients (71%), there was an increase in C3 concentrations with a decrease in anti-C3 levels during the course of treatment. C4 levels in patients positive for anti-C3 (median: 0.138 g/l, IQR: 0.073–0.211) were also lower than C4 levels in negative ones (median: 0.274 g/l, IQR: 0.145–0.338) (Mann-Whitney, *P* = 0.0394) (Figure 2c). The same difference was present in the C4 levels found among all tested samples in patients monitored in dynamics: C4 levels in samples positive for anti-C3 (median: 0.173 g/l, IQR: 0.108–0.279) was lower than C4 levels in negative ones (median: 0.240 g/l, IQR: 0.170–0.310) (*P* = 0.0013) (Figure 2d). However, no such differences were found between C3 and C4 levels in the positive and the negative patients for anti-C4 (not shown).

### Anti-C3 and to a Lesser Extent Anti-C4 Autoantibodies are Associated With Anti-dsDNA Levels

Comparative analysis of anti-dsDNA levels revealed a difference between the patients positive for anti-C3 (median: 34.1 U/ml, IQR: 16.5–84.4) and levels in negative ones (median: 10.0 U/l, IQR: 6.3–17.4; Mann-Whitney *P* < 0.0001), at cross-section (Figure 2e), as well as when the samples were compared in dynamics (median: 35.7 U/ml, IQR: 23.5–69.8) for the anti-C3 positive versus median: 14.1 U/ml, IQR: 8.3–26.6 for the anti-C3 negative samples; Mann-Whitney *P* < 0.0001) (Figure 2f). Moreover, there was a moderate positive correlation between anti-C3 levels and anti-dsDNA levels among patients with LN (Spearman r = 0.4656, *P* < 0.0001) at cross-section and in dynamics (Spearman r = 0.4790, *P* < 0.0001).

There was no significant difference between levels of anti-dsDNA in patients positive for anti-C4 (median: 21.5 U/ml, IQR: 10.6–70.7) and levels in negative ones (median: 11.2 U/ml, IQR: 7.3–23.6) (Mann-Whitney, *P* = 0.0890 (Figure 2g). The difference became significant only when all samples were considered (median: 28.6 U/ml, IQR: 16.2–67.9 for the anti-C4 positive vs. 18.8 U/ml, IQR: 9.9–37.7 for the negative ones; Mann-Whitney test, *P* = 0.0139) (Figure 2h). There was only a weak positive correlation between anti-C4 levels and anti-dsDNA levels among patients with LN (Spearman r = 0.2382, *P* = 0.0424) at cross-section and in dynamics (Spearman r = 0.2801, *P* < 0.0001).

### Elevated Anti-C3 Autoantibodies Correlate With Proteinuria

**Figure 3:**
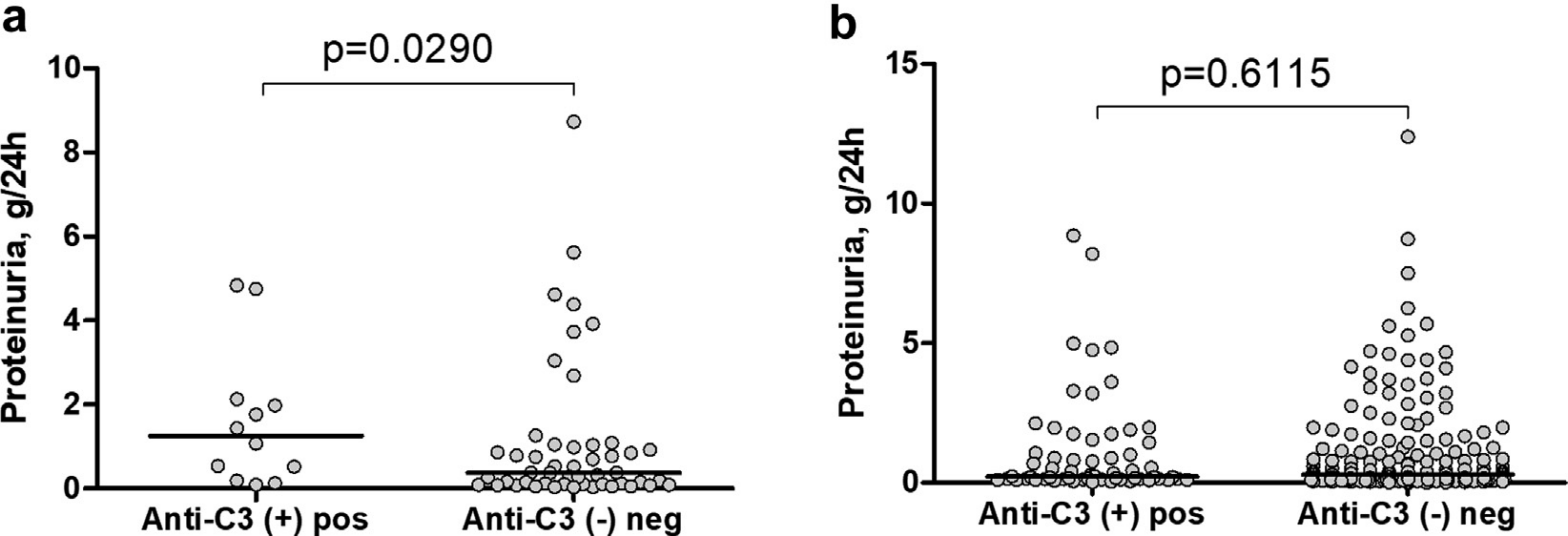
Association of anti-C3 autoantibodies with proteinuria. (a) Proteinuria is higher in anti-C3 positive patients than in negative ones at cross-sectional analysis. (b) Proteinuria did not differ between anti-C3 positive and anti-C3 negative samples in dynamic, Mann-Whitney test. neg, negative; pos, positive.

Median level of proteinuria in patients positive for anti-C3 (1.11 g/24 h, IQR: 0.13–4.15) is higher than the negative ones (0.35 g/24 h, IQR: 0.08–0.10; Mann-Whitney, *P* = 0.0290) at cross section analysis (Figure 3a); however, there was no difference in proteinuria between anti-C3 positive (0.23 g/24 h, IQR: 0.13–1.49) and anti-C3 negative (0.29 g/24h, IQR: 0.10–1.18; Mann-Whitney, *P* = 0.6115) samples in dynamic (Figure 3b). No difference was observed for anti-C4 (0.35 g/24 h, IQR: 0.06–4.46 for anti-C4 positive vs. 0.52 g/24 h, IQR: 0.11–1.18 for the negative ones; Mann-Whitney, *P* = 0.7845).

Stratification of the patients as double positive, single positive for anti-C3 or anti-C4 or double negative did not reveal a difference from anti-C4 status on proteinuria in the 2 groups with respect to anti-C3 status (2-way analysis of variance, F = 2.20, *P* = 0.1417) and only anti-C3 statistically affects proteinuria (F = 5.08, *P* = 0.0270).

At cross-section, there was no significant association of the anti-C3 or anti-C4 titers with the urinary sediment. Nevertheless, in dynamics, the median level of anti-C3 in patients with active urinary sediment (0.693 NU [IQR: 0.278–1.875]) was higher than median level of anti-C3 in patients with nonactive urinary sediment (0.477 NU [IQR: 0.167–0.856]; Mann-Whitney, *P* = 0.0006). Similarly, the median level of anti-C4 in patients with active urinary sediment (0.554 NU [IQR: 0.348–0.996]) was higher than the median level of anti-C4 in patients with nonactive urinary sediment (0.427 NU [IQR: 0.232–0.689]; Mann-Whitney, *P* = 0.0006).

### Anti-C3 and Anti-C4 Associate With Class IV LN and the Histological Index of Activity

**Figure 4:**
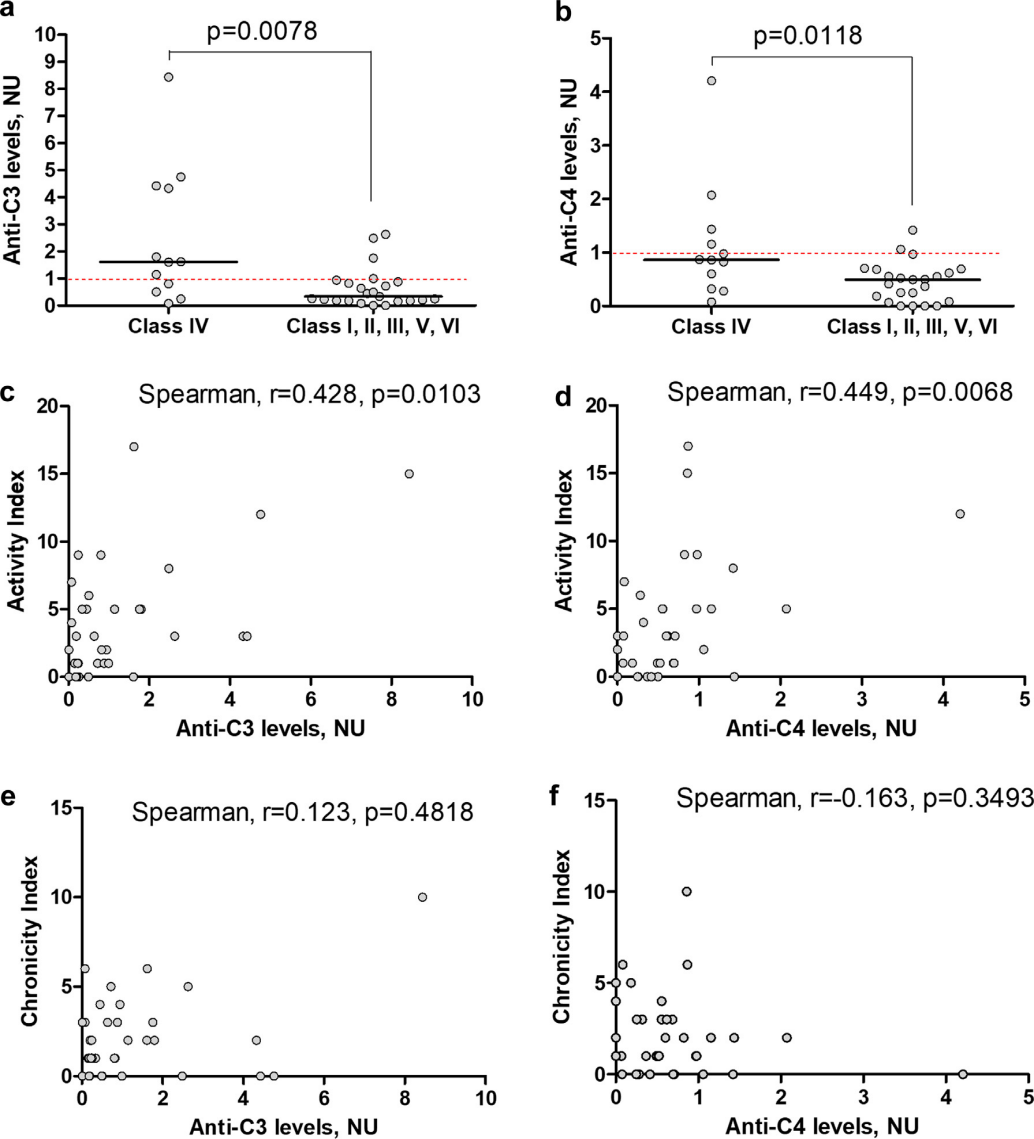
Association between anti-C3 and anti-C4 antibodies and histology class of LN and histological indexes of activity and chronicity. (a) Anti-C3 and the class of LN. (b) anti-C4 and the class of LN. Mann-Whitney test. (c) Anti-C3 and the activity index, (d) anti-C4 and the activity index. (e) Anti-C3 and the chronicity index, and (f) anti-C4 and the chronicity index. Spearman correlation analysis. LN, lupus nephritis.

There is a significant difference between anti-C3 or anti-C4 levels in patients with class IV compared to all other classes of LN (Mann-Whitney *P* = 0.0078 and *P* = 0.0118, respectively) (Figure 4a and b).

Moreover, there was a positive correlation between anti-C3 and anti-C4 and the histological index of activity (Spearman r = 0.428, *P* = 0.0103; and r = 0.449, *P* = 0.0068, respectively, Figure 4c and d). These correlations were driven by the association of both anti-C3 and anti-C4 with subendothelial deposits (wire loops) as well as with the interstitial inflammation (immune cell infiltration) for anti-C3 and the endocapillary hypercellularity for anti-C4 (Table 2). On the contrary, there were no significant correlations between anti-C3 and anti-C4 and chronicity index and its individual parameters (Figure 4e and f, Table 2) and no association was found between the immunofluorescence markers for immunoglobulins and complement, and autoantibody positivity.

**Table 2:** Comparative analysis of anti-C3 and anti-C4 levels, according to presence and absence of histological signs of activity and chronicity in patients with LN

Histological features	Anti-C3 levels, NU (median [IQR])		*P* value	Anti-C4 levels, NU (median [IQR])		*P* value
	Presence	Absence		Presence	Absence	
Endocapillary hypercellularity	0.761 (0.242–1.786)	0.250 (0.182–0.939)	0.160	0.657 (0.365–0.976)	0.253 (0.000–0.498)	0.019
Glomerular leucocyte infiltration	2.485 (0.500–4.758)	0.564 (0.193–1.491)	0.118	1.421 (0.283–4.206)	0.539 (0.201–0.850)	0.166
Subendothelial deposits “Wire loops”	1.621 (0.973–3.277)	0.470 (0.188–1.146)	0.036	1.155 (0.923–3.139)	0.496 (0.160–0.699)	0.002
Fibrinoid necrosis	0.636 (0.189–2.485)	0.606 (0.210–1.491)	0.749	0.824 (0.322–0.979)	0.496 (0.104–0.693)	0.073
Cellular crescents	1.795 (0.849–6.598)	0.496 (0.201–1.030)	0.073	0.867 (0.472–3.139)	0.511 (0.233–0.737)	0.070
Interstitial inflammation (infiltration)	1.383 (0.419–3.053)	0.250 (0.182–0.811)	0.009	0.643 (0.084–0.893)	0.494 (0.251–0.843)	0.649
Glomerular sclerosis	0.636 (0.182–1.621)	0.652 (0.210–2.150)	0.766	0.554 (0.253–0.858)	0.609 (0.120–1.040)	0.868
Fibrous crescents	1.621 (0.477–5.117)	0.496 (0.201–1.259)	0.220	0.858 (0.386–1.470)	0.511 (0.233–0.861)	0.229
Tubular atrophy	0.720 (0.205–1.621)	0.496 (0.203–1.633)	1.000	0.524 (0.086–0.687)	0.648 (0.304–1.040)	0.161
Interstitial fibrosis	0.939 (0.542–2.193)	0.292 (0.182–1.146)	0.079	0.600 (0.219–0.863)	0.496 (0.208–0.993)	0.878

IQR, interquartile range; LN, lupus nephritis.

Anti-C3 and andti-C4 status correlate with histological features of activity but of chronicity of LN.

### The Anti-C3 Status of Patients Significantly Determines the Activity of LN and the Need for Immunosuppression

**Figure 5:**
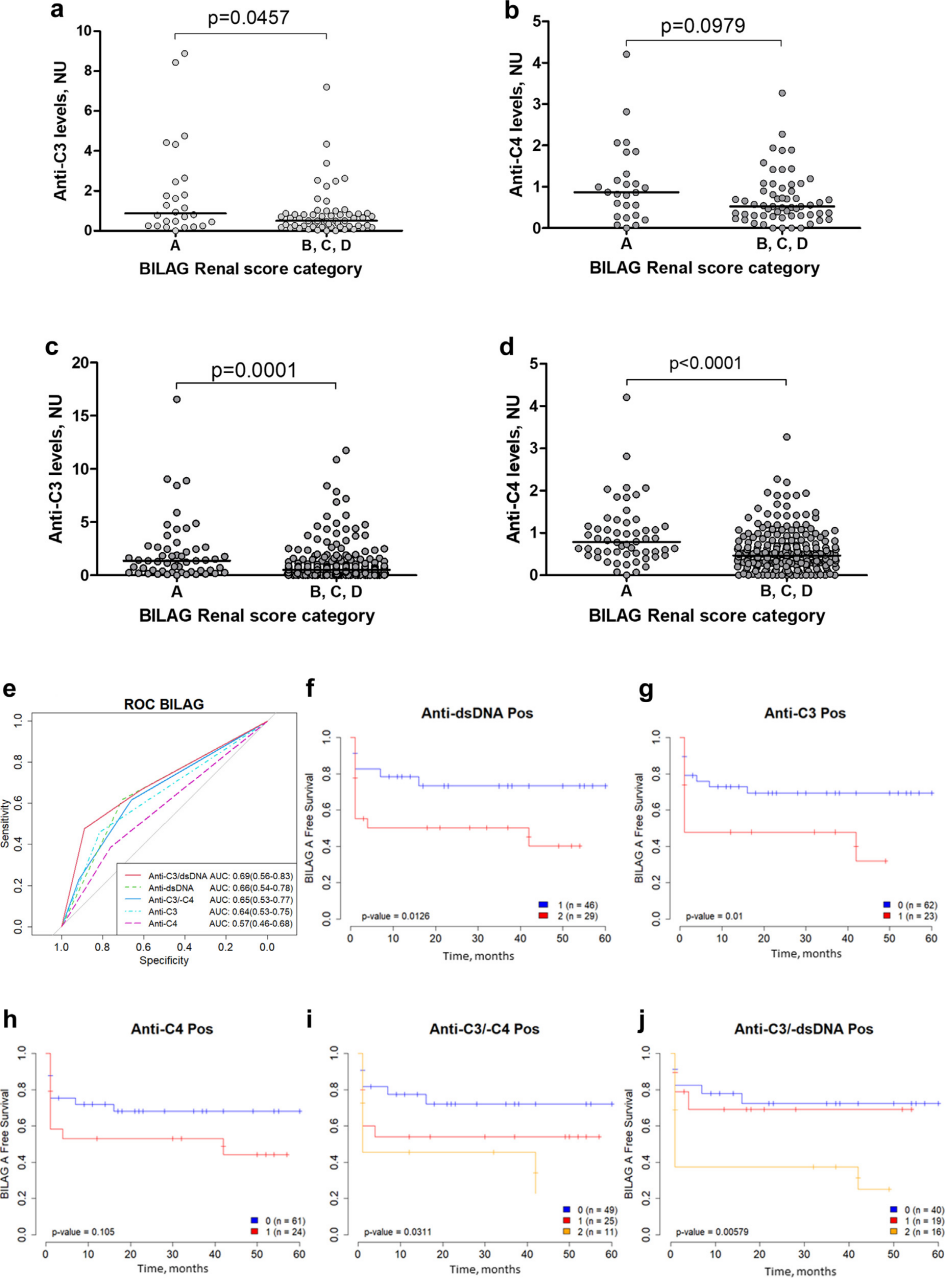
Association of anti-C3 and anti-C4 autoantibodies with activity of LN and the need for immunosuppressive therapy. Association of BILAG Renal score category with (a) anti-C3 in cross-section, (b) anti-C4 in cross-section, (c) anti-C3 in dynamic, (d) anti-C4 in dynamic (Mann-Whitney test). (e) receiver operating characteristic curves from univariate logistic models predicting current BILAG A status for all the autoantibodies at visit 1. BILAG A-free survival Kaplan-Meier curves according to autoantibody positivity at visit 1 for (f) anti-dsDNA, (g) anti-C3, and (h) anti-C4. Blue curves are for the negative patients (0) and red for the positive (1). BILAG A-free survival Kaplan-Meier curves according to autoantibody positivity at visit 1 for (i) double anti-C3/anti-C4, and (j) double anti-C3/anti-dsDNA. Blue curves are for double negative patients (0), red for single positive (1), and orange for double positive (2). Log-rank test *P*-value is shown. BILAG, British Isles Lupus Assessment Group; LN, lupus nephritis; neg, negative; pos, positive.

Cross-section analysis revealed that the median level of anti-C3 in patients with BILAG A (0.871 NU, IQR: 0.254–2.502) is higher than the one of all other BILAG categories (0.508 NU, IQR: 0.205–0.871) (Mann-Whitney *P* = 0.0457, Figure 5a) but not of the median level of anti-C4 (Mann-Whitney *P* = 0.0979, Figure 5b). In dynamics, patients’ distribution demonstrates that positivity for anti-C3 significantly determined the presence of BILAG A with an odds ratio (OR) of 3.477 (95% confidence interval [CI]: 1.656–7.303) (Fisher exact test, *P* = 0.0013). Median level of anti-C3 among all samples tested in dynamics with category A according to BILAG (1.356 NU [IQR: 0.326–2.644]) was higher than median level of anti-C3 in samples with all other BILAG categories (0.500 NU, IQR: 0.184–0.985) (Mann-Whitney *P* = 0.0001, Figure 5c).

For the anti-C4 IgG, the distribution demonstrated that the positivity did not significantly determine the presence of BILAG A (Fisher exact test *P* = 0.0879). Nevertheless, median level of anti-C4 among all samples tested in dynamics with BILAG A (0.785 NU, IQR: 0.501–1.158) was higher than the median level of anti-C4 in samples with all other categories (0.464 NU, IQR: 0.275–0.708) (Mann-Whitney, *P* < 0.0001, Figure 5d).

The positivity for both anti-C3 and anti-C4 significantly determined the presence of BILAG A in patients with LN and the double anti-C3 and anti-C4 positivity significantly determined the presence of BILAG A with an OR of 9.000 (95% CI: 3.708–21.840) (Fisher exact test, *P* < 0.0001).

The comparison of autoantibodies as biomarkers to predict present BILAG A was performed with receiver operating characteristic curve analyses (Figure 5e). Anti-C3 were better markers to predict BILAG category A at first visit with area under the curve 0.64 (0.53–0.75), OR (95% CI) of 3.74 (1.37–10.51), (*P* = 0.011), than anti-C4, with area under the curve 0.57 (0.46–0.68), OR (95% CI) of 2.01 (0.74–5.44), (*P* = 0.168). Anti-C3 had similar predictive value as classical biomarker such as anti-dsDNA (area under the curve 0.66 (0.54–0.78), OR (95% CI) 3.86 (1.37–11.54), (*P* = 0.012) and the combination of the anti-C3/anti-dsDNA.

Importantly, the positivity for anti-C3 at first visit predicted a subsequent progression of the disease to BILAG A (log-rank test, *P* = 0.01), (univariate Cox analysis, hazard ratio 2.67 (1.28–5.57), *P* = 0.00857) similar to dsDNA, (Figure 5f and g), whereas anti-C4 positivity showed only a trend (log-rank test, *P* = 0.105; hazard ratio 1.86 (0.88–3.91), *P* = 0.103) (Figure 5h). The combination of the 2 parameters was predictive (log-rank test, *P* = 0.0311, hazard ratio for single positive 2.14 (0.93–4.95), (*P* = 0.075) and hazard ratio for double positive 3.15 (1.32–8.84), (*P* = 0.0114), (Figure 5i); however, the combination of these 2 biomarkers did not add much value compared to the anti-C3 alone. Finally, the combination of anti-C3 and anti-dsDNA was the best predictor, hazard ratio double positive 4.04 (1.67–9.74), (*P* = 0.005) (Figure 5j).

## Discussion

Here, we describe presence of elevated anti-C3 and anti-C4 autoantibodies in patients with LN and their evolution over time. Even though double positive patients were detected, the reactivity against C3 was independent of that against C4. Anti-C3 showed stronger clinical correlations, compared to anti-C4, and were associated with hypocomplementemia, anti-dsDNA, as well as with class IV LN, histological index of activity and BILAG A using cross-section analyses and in dynamics. In dynamics, positivity for anti-C3 at first sampling alone and especially when combined with anti-dsDNA predicted future aggravation of the disease, measured by a transition to BILAG A. Therefore, anti-C3/C3b are useful biomarkers for disease activity, which, combined with anti-dsDNA are strong predictors of future flares.

We detected anti-C3 and anti-C4 in 27% and 28%, respectively of the patients with LN in a cross-section analysis. This frequency is consistent with previous reports^1^^,^^3^, ^4^, ^5^, ^6^, ^7^, ^8^, ^9^, ^10^, ^11^, ^12^, ^13^, ^14^, ^15^ and higher compared to C3G (about 6%),^18^ a disease for which the measurement of these autoantibodies is recommended. Moreover, anti-C3 found in C3G displays distinct properties compared to LN. Moreover, the LN anti-C3 IgG have clear-cut functional consequences^1^^,^^3^, ^4^, ^5^, ^6^, ^7^, ^8^, ^9^, ^10^, ^11^, ^12^, ^13^, ^14^, ^15^ and inhibit the interaction of C3b with Factor H and CR1, whereas the few tested anti-C3 in C3G had heterogeneous effect and inhibited the action of CR1 but not Factor H.^18^ In C3G, functionally relevant autoantibodies are C3 nephritic factors and anti-Factor B IgG,^19^, ^20^, ^21^ but these are absent in LN.^5^ This suggests that the immune response against the C3 convertase constituents is different in C3G and LN.

Systemic lupus erythematosus is characterized by dysregulated antibody response, which is often cross-reactive and polyreactive.^22^ Probing the reactivity of antigen-specific anti-C3 and anti-C4, purified from double-positive plasma, and the prevalent detection of either anti-C3 alone or anti-C4 alone suggest that in most cases these antibodies have unique specificity and likely arise from different B cell clones. In 1 case, we detected cross-reactive antibodies, suggesting that such cross-reactivity is also possible in LN. One of the epitopes, TIYTPG on C3b and QPIYNPGQR on C4b lays in an area, where the regulatory molecules FH and CD46 bind. The structure of the complex of CD46 with C4b is not solved, but if the binding is similar to C3b, one can hypothesize perturbation of the binding of the regulators, as we have already described for anti-C3.^5^ The relative independence of the 2 autoantibodies prompted us to explore their clinical relevance.

The accurate differentiation between active nephritis and chronic kidney damage holds pivotal importance.^1^ The assessment of disease activity and kidney damage relies on biopsy, as well as clinical measurements of kidney function, urinary findings. It is noteworthy that renal biopsies are infrequent, and clinical measures are constrained in reflecting intrarenal injury with precision. Therefore, the identification of novel biomarkers for evaluating LN activity emerges as a critical unmet requirement within LN management.

Anti-C3 positive patients had C3 and C4 consumption both at cross-section and in dynamics, consistent with their capacity to dysregulate the cascade.^4^^,^^5^ On the contrary, anti-C4 positive patients did not differ in their C3 and C4 plasma concentrations, suggesting lack of direct effect to dysregulate or overactivate the cascade. Moreover, significantly higher anti-C3 in patients with interstitial inflammation is probably an expression of the biological effect of these antibodies, causing overactive alternative pathway and leading to inflammatory renal damage in patients with LN. This, along with the severity of LN, is important for the prognosis of this disease.

Positive anti-C3 reactivity was found to be associated with proteinuria and elevated anti-dsDNA levels, which are classical markers indicative of the severity and immune activity in LN. This observation aligns with previous findings.^4^, ^5^, ^6^ Conversely, there was no significant difference in anti-C4 reactivity. However, both anti-C3 and anti-C4 titers were notably higher in the class IV LN than in other classes. These elevated titers correlated with the histological activity index but not with the chronicity index.

To determine which of the 2 autoantibodies was more relevant in determining LN activity and the requirement for immunosuppressive therapy, we employed the BILAG for disease activity assessment. Remarkably, anti-C3 showed superior performance. Its titers were substantially elevated in cases with BILAG A compared to other categories, both in cross-sectional and follow-up. Moreover, anti-C3 exhibited enhanced discriminatory power as a marker for active disease (BILAG A) in receiver operating characteristic curve analysis compared to anti-C4. In addition, the presence of anti-C3 in the initial sample significantly predicted the future progression to BILAG A, better than anti-C4. Combining anti-C3 with dsDNA out-perform each marker alone. Consequently, anti-C3 stands out as an effective LN disease activity marker, especially when combined with anti-dsDNA. Anti-C3 IgG appear to be more relevant for LN compared to C3G both in frequency and clinical significance and their measurement in LN could be useful to predict future flairs.

In conclusion, even though both anti-C3 and anti-C4 were detected in a subset of patients with LN, only anti-C3 exhibited notable clinical relevance, likely due to their functional consequences. This work underscores the need for further studies on the potential clinical utility of anti-C3 autoantibodies in combination with anti-dsDNA as biomarkers of disease activity in LN.

## Disclosure

All the authors declared no competing interests.

## Supplementary Material

Supplementary File (PDF)

**Table S1.** Comparison of the demographic, clinical, immunological, and histological characteristics of patients with lupus nephropathy according to anti -C3 and anti - C4 status at first sampling.

## References

[1] Birmingham D.J., Merchant M., Waikar S.S., Nagaraja H., Klein J.B., Rovin B.H. (2017). Biomarkers of lupus nephritis histology and flare: deciphering the relevant amidst the noise. Nephrol Dial Transplant.

[2] Bao L., Cunningham P.N., Quigg R.J. (2015). Complement in lupus nephritis: new perspectives. Kidney Dis (Basel).

[3] Vasilev V.V., Radanova M., Lazarov V.J., Dragon-Durey M.A., Fremeaux-Bacchi V., Roumenina L.T. (2019). Autoantibodies against C3b-functional consequences and disease relevance. Front Immunol.

[4] Tao J., Song D., Liu X.L., Yu F., Zhao M.H. (2020). Circulating anti-C3b IgG in lupus nephritis: a large cohort study. Clin Immunol.

[5] Vasilev V.V., Noe R., Dragon-Durey M.A. (2015). Functional characterization of autoantibodies against complement Component C3 in patients with lupus nephritis. J Biol Chem.

[6] Birmingham D.J., Bitter J.E., Ndukwe E.G. (2016). Relationship of circulating anti-C3b and anti-C1q IgG to lupus nephritis and its flare. Clin J Am Soc Nephrol.

[7] Durand C.G., Burge J.J. (1984). A new enzyme-linked immunosorbent assay (ELISA) for measuring immunoconglutinins directed against the third component of human complement. Findings in systemic lupus erythematosus. J Immunol Methods.

[8] Kenyon K.D., Cole C., Crawford F. (2011). IgG autoantibodies against deposited C3 inhibit macrophage-mediated apoptotic cell engulfment in systemic autoimmunity. J Immunol.

[9] Kianmehr N., Khoshmirsafa M., Shekarabi M. (2021). High frequency of concurrent anti-C1q and anti-dsDNA but not anti-C3b antibodies in patients with lupus Nephritis. J Immunoassay Immunochem.

[10] Lachmann P.J. (1967). Conglutinin and immunoconglutinins. Adv Immunol.

[11] Nilsson B., Ekdahl K.N., Sjoholm A., Nilsson U.R., Sturfelt G. (1992). Detection and characterization of immunoconglutinins in patients with systemic lupus erythematosus (SLE): serial analysis in relation to disease course. Clin Exp Immunol.

[12] Nilsson B., Ekdahl K.N., Svarvare M., Bjelle A., Nilsson U.R. (1990). Purification and characterization of IgG immunoconglutinins from patients with systemic lupus erythematosus: implications for a regulatory function. Clin Exp Immunol.

[13] Omidi F., Khoshmirsafa M., Kianmehr N. (2022). Comparison of circulating miR-148a and miR-126 with autoantibodies as biomarkers of lupus nephritis in patients with SLE. J Immunoassay Immunochem.

[14] Pradhan V.D., Khadilkar P.V., Nadkar M.Y. (2021). Impact of autoantibodies to complement components on the disease activity in SLE. J Assoc Phys India.

[15] Radanova M., Roumenina L.T., Vasilev V. (2021). Detection of anti-C3b autoantibodies by ELISA. Methods Mol Biol.

[16] Hay E.M., Bacon P.A., Gordon C. (1993). The BILAG index: a reliable and valid instrument for measuring clinical disease activity in systemic lupus erythematosus. Q J Med.

[17] Weening J.J., D’Agati V.D., Schwartz M.M. (2004). The classification of glomerulonephritis in systemic lupus erythematosus revisited. J Am Soc Nephrol.

[18] Marinozzi M.C., Roumenina L.T., Chauvet S. (2017). Anti-factor B and anti-C3b autoantibodies in C3 glomerulopathy and Ig-associated membranoproliferative GN. J Am Soc Nephrol.

[19] Chauvet S., Berthaud R., Devriese M. (2020). Anti-factor B antibodies and acute postinfectious GN in children. J Am Soc Nephrol.

[20] Corvillo F., Okroj M., Nozal P., Melgosa M., Sánchez-Corral P., López-Trascasa M. (2019). Nephritic factors: an overview of classification, diagnostic tools and clinical associations. Front Immunol.

[21] Marinozzi M.C., Chauvet S., Le Quintrec M. (2017). C5 nephritic factors drive the biological phenotype of C3 glomerulopathies. Kidney Int.

[22] Zhang J., Jacobi A.M., Wang T., Berlin R., Volpe B.T., Diamond B. (2009). Polyreactive autoantibodies in systemic lupus erythematosus have pathogenic potential. J Autoimmun.

