# Supplementary material for: Clinical Relevance of Anti-C3 and Anti-C4 Autoantibodies in Lupus Nephritis
Source: Kidney Int Rep. 2024 Feb 2;9(5):1429–40. doi: 10.1016/j.ekir.2024.01.052 (PMC11068950; doi:10.1016/j.ekir.2024.01.052)
Supplement: Supplementary File (PDF) [file mmc1.pdf]

Supplementary Table 1. Comparison of the demographic, clinical, immunological, and histological characteristics of patients with lupus nephropathy according to anti-C3 and anti-C4 status at first sampling.

Characteristic	Anti-C3 Negative (N=62)	Anti-C3 Positive (N=23)	p-value	Anti-C4 Negative (N=61)	Anti-C4 Positive (N=24)	p-value
**Age**<br>Median yr. (IQR)	46.5 (38.0-58.0)	34.0 (29.0-39.0)	**0.001**	44.0 (35.0-57.0)	39.0 (35.0-54.8)	0.551
**Serum Creatinine**<br>Median µmol/L (IQR)	70.0 (58.5-90.8)	64.0 (59.0-77.0)	0.739	69.0 (59.0-93.0)	65.0 (57.5-77.5)	0.278
**eGFR**<br>Median ml/min/1,73 sqm (IQR)	86.0 (64.0-110.5)	94.0 (81.0-118.0)	0.221	88.0 (64.0-113.0)	93.0 (78.0-107.5)	0.574
**Proteinuria**<br>Median g/l (IQR)	0.3 (0.1-1.0)	1.1 (0.1-4.2)	**0.032**	0.5 (0.1-1.1)	0.3 (0.1-4.3)	0.781
**ESR**<br>Median mm/h (IQR)	19.0 (10.0-30.0)	20.0 (16.0-50.0)	0.072	20.0 (10.0-29.0)	32.0 (11.5-52.5)	0.058
**Hb**<br>Median g/L (IQR)	134.5 (122.2-145.0)	126.0 (115.0-143.0)	0.197	134.0 (121.0-146.0)	127.5 (116.5-137.5)	0.226
**Erytrocyte count**<br>Median T/L (IQR)	4.5 (4.1-4.8)	4.6 (4.1-4.9)	0.840	4.5 (4.2-4.9)	4.2 (4.1-4.8)	0.485
**WBC count**<br>Median G/L (IQR)	6.7 (5.7-8.6)	6.3 (4.9-7.6)	0.334	6.7 (5.7-8.7)	6.3 (4.9-7.6)	0.167
**Platelet count**<br>Median G/L (IQR)	256.0 (203.2-308.0)	236.0 (206.0-293.0)	0.818	237.0 (210.0-300.0)	259.0 (194.2-359.5)	0.513
**Total Protein**<br>Median g/L (IQR)	71.0 (68.0-75.0)	66.0 (60.0-73.0)	**0.029**	70.0 (66.0-74.0)	71.0 (65.0-76.0)	0.467
**Serum Albumin**<br>Median g/L (IQR)	42.0 (39.0-43.8)	38.0 (31.0-41.0)	**<0.001**	42.0 (38.0-44.0)	39.5 (34.0-41.8)	**0.022**
**Uric Acid**<br>Median µmol/L (IQR)	311.0 (245.5-408.8)	392.0 (308.2-426.0)	**0.045**	339.0 (251.0-424.0)	312.0 (250.5-367.0)	0.274
**Total Cholesterol**<br>Median mmol/L (IQR)	6.0 (5.2-6.7)	5.7 (4.7-7.1)	0.653	6.1 (5.3-7.0)	5.1 (4.5-6.4)	**0.029**
**Triglycerides**<br>Median mmol/L (IQR)	1.4 (1.1-1.8)	1.8 (1.5-2.2)	**0.011**	1.4 (1.1-1.9)	1.7 (1.4-2.1)	0.084
**CRP**<br>Median mg/dL (IQR)	0.3 (0.1-0.5)	0.3 (0.2-0.6)	0.699	0.3 (0.1-0.5)	0.2 (0.2-0.6)	0.846
**C3 complement**<br>Median g/L (IQR)	1.2 (1.0-1.5)	1.0 (0.6-1.1)	**<0.001**	1.2 (1.0-1.5)	1.0 (0.6-1.2)	**0.039**
**C4 complement**<br>Median g/L (IQR)	0.3 (0.1-0.3)	0.1 (0.1-0.2)	**<0.001**	0.3 (0.1-0.3)	0.2 (0.1-0.2)	**0.013**
**ANA titer** (N < 1:80)<br>Median (IQR)	160.0 (80.0-640.0)	960.0 (640.0-1280.0)	**<0.001**	160.0 (80.0-640.0)	640.0 (320.0-1280.0)	**0.009**
**Anti-dsDNA**<br>Median U/mL (IQR)	10.0 (6.5-17.0)	32.0 (16.5-80.0)	**<0.001**	11.2 (7.5-23.5)	21.5 (13.4-64.9)	0.088
**Activity index** (0 – 24)<br>Median (IQR)	1.5 (0.8-4.2)	5.0 (3.0-9.0)	**0.011**	3.0 (1.0-5.0)	5.0 (2.8-7.2)	0.289
**Chronicity index** (0 – 12)<br>Median (IQR)	1.0 (0.8-3.0)	2.0 (1.5-5.0)	0.225	2.0 (1.0-3.0)	1.0 (0.0-2.0)	0.160
